# Letter on Oral Health of Stone Mine Workers

**DOI:** 10.1016/j.shaw.2014.12.007

**Published:** 2015-01-23

**Authors:** Anindya Kumar Ganguli

**Affiliations:** Occupational Health Service, Bharat Heavy Electricals Limited, Tiruchirapalli, India

To the Editor

In the article “Oral Health of Stone Mine Workers of Jodhpur City, Rajasthan, India” recently published in *SH@W*, the authors conclude that the oral health of stone mine workers is in a poor state [1]. This conclusion is misleading, because it tends to suggest that stone mining as an occupation presents hazards which lead to poor oral health. This has not been established anywhere in the article.

First, there is no reason to assume an occupational association between stone dust and dental caries. In the Discussion, the authors state that “an association between attrition and stone mine jobs has been established” yet do not suggest what could be the linkage between dust and poor oral health, considering that other factors like age and years of use of tobacco and alcohol have not been controlled.

Second, although the authors state that the habit of alcohol consumption and tobacco use leads to deterioration of the workers' oral health, no comparisons were made with a control group with the same habits and occupation. Without the benefit of such a comparison, is it correct to conclude that stone mining leads to poor oral health?

Third, the values of the decayed, missing, and filled teeth (DMFT) index for different age groups in marble mine laborers (3.13) [2] or transport workers (5.02) [3] are higher than reported in this article (2.89). Even the age-specific highest value reported in this article (3.59 for the 37–46 year age group) is lower than that reported for marble mine workers (4.0 seen in the 45–54 year age group). Hence DMFT, at least, is better in stone mine workers than marble mine workers or transport workers. Interestingly, Table 2 in [1] shows the DMFT index (mean) to be 3, while the text says it is 2.89.

Hence, this article reports the point prevalence of oral health indices in stone mine workers in Rajasthan, India and should not attempt to relate these findings to occupational hazards of stone mining.

## Conflicts of interest

The author declares no conflicts of interest.
